# The Rising Challenge of Stroke in Iraq and the Importance of Endovascular Centers

**DOI:** 10.7759/cureus.74174

**Published:** 2024-11-21

**Authors:** Ali Alshalchy, Mustafa F Kadhim, Mostafa H Algabri, Saleh Saleh, Mustafa Ismail

**Affiliations:** 1 Department of Surgery, University of Baghdad, College of Medicine, Baghdad, IRQ; 2 Department of Neurology, Al-Yarmouk Teaching Hospital, Baghdad, IRQ; 3 Department of Surgery, Baghdad Teaching Hospital, Baghdad, IRQ

**Keywords:** endovascular centers, healthcare infrastructure, mechanical thrombectomy, stroke care, treatment accessibility

## Abstract

Stroke is a critical public health issue in Iraq, where the rising incidence is met with insufficient specialized care, often leading to severe disability or mortality. This editorial highlights the urgent need for establishing endovascular centers across the country to address these gaps. Endovascular interventions, such as mechanical thrombectomy, have proven effective in reducing stroke-related mortality and long-term disability when administered within a narrow treatment window. However, Iraq faces numerous challenges, including limited resources, infrastructure deficits, and a shortage of trained specialists, compounded by socio-political factors that affect healthcare delivery. Iraq at this moment lacks a dedicated stroke endovascular center. By investing in endovascular centers and addressing the systemic challenges, Iraq can align its stroke care with global standards, offering improved outcomes for stroke patients and reducing the nation’s overall healthcare burden.

## Editorial

Background

Stroke has become an urgent public health crisis in Iraq, with cases and associated disabilities steadily increasing each year. Despite advancements in stroke care worldwide, Iraq struggles with limited access to timely and effective treatment options, which often results in severe disability or death for many patients [1]. Key challenges in addressing stroke care include a lack of specialized stroke centers, infrastructure limitations, and ongoing security issues. Years of conflict have impacted Iraq's healthcare infrastructure, resulting in resource shortages and limited access to advanced care. Furthermore, many patients face delays in reaching medical facilities, especially in rural areas where healthcare resources are even scarcer [1].

Endovascular centers, which offer minimally invasive procedures like mechanical thrombectomy, represent a promising solution for improving stroke outcomes. By rapidly clearing blockages in major arteries, these procedures can reduce long-term disability and increase survival rates when performed within critical hours after a stroke [2]. Establishing such centers in Iraq would not only align with global best practices but also bring lifesaving treatments closer to more patients across the country [2]. This editorial highlights the pressing need for endovascular centers in Iraq, examining the societal and healthcare barriers that must be addressed to make these advanced treatments accessible and effective.

Understanding the need for endovascular centers in Iraq

In 2019, stroke incidence rates in Iraq varied from 196.2 to 218.3 per 100,000 persons, as reported by Global Burden of Disease 2019 Stroke Collaborators [3]. This is largely due to a high prevalence of stroke risk factors, such as hypertension, diabetes, and smoking [1]. Despite the high need, stroke care facilities remain limited, with few hospitals equipped to perform advanced procedures such as mechanical thrombectomy. Consequently, many patients experience significant delays in receiving necessary care, which can lead to poorer outcomes [1]. In Iraq, geographic and logistical limitations often prevent patients from reaching specialized centers within the critical treatment window of six hours, leaving many without the chance to receive life-saving interventions [1].

According to Ismail et al., neurosurgeons in Iraq encounter significant barriers related to inadequate healthcare resources and delayed presentations, due to complex social and political challenges [1]. Many patients arrive at medical facilities in severe conditions, and even if the prognosis is poor, families may demand extended care, which further depletes already limited resources [1]. Establishing endovascular centers across the country would not only improve access but also increase the likelihood of timely interventions, directly reducing disability and mortality rates among stroke patients.

Efficacy of endovascular treatment in improving stroke outcomes

Endovascular interventions, including thrombectomy, have shown to be highly effective in reducing mortality and long-term disability among stroke patients, particularly when performed within hours of symptom onset. Studies indicate that patients treated at high-volume endovascular centers experience faster intervention times and better recovery rates due to the availability of specialized equipment and trained professionals. A review of Austria’s nationwide stroke care system highlights the effectiveness of endovascular care in improving survival rates and quality of life for patients, particularly when these services are readily accessible [2].

In Iraq, where stroke patients often have limited access to specialized stroke treatment, establishing endovascular centers could significantly improve outcomes [4]. The presence of these centers would allow for the prompt intervention necessary to minimize brain damage in acute stroke cases, bridging the gap between Iraq’s current healthcare resources and the international standard for stroke care [4]. Building these centers is a pivotal step toward establishing a more equitable and effective healthcare system in Iraq.

Societal challenges impacting neurosurgeons in Iraq

Neurosurgeons in Iraq operate under exceptionally high-stress conditions, facing risks not only due to the complex nature of their work but also from external social pressures. According to Ismail et al., neurosurgeons in Iraq encounter substantial threats from patients’ families if treatment outcomes are unfavorable [1]. This environment can make doctors hesitant to take on high-risk cases or pursue advanced specialties such as vascular neurosurgery, ultimately limiting the availability of skilled professionals in Iraq [1].

These social challenges compound the country’s healthcare limitations, as neurosurgeons may choose to emigrate in search of safer, more supportive working environments. The shortage of qualified neurosurgeons in Iraq undermines the healthcare system’s ability to provide specialized care, particularly in fields like endovascular neurosurgery, where precision and experience are paramount. Addressing these social pressures is essential for retaining talent within Iraq and ensuring that endovascular centers can operate with a full team of skilled practitioners.

Impacts on healthcare infrastructure and retention of specialists

The “brain drain” phenomenon has become increasingly pronounced in Iraq, as skilled neurosurgeons opt to practice abroad where the risks to personal safety are lower, and professional resources are more abundant. This trend has limited Iraq’s ability to expand endovascular capabilities, as the loss of specialized neurosurgeons creates a critical shortage in key areas of stroke care. According to Ismail et al., the emigration of doctors due to societal pressures and lack of resources has a detrimental effect on healthcare delivery in Iraq [1]. The departure of skilled professionals not only affects stroke care but also impacts the broader medical infrastructure, limiting the capacity to develop comprehensive, specialized treatment networks [1].

Developing endovascular centers in Iraq would, therefore, necessitate not only infrastructure investment but also a focus on improving working conditions and societal support for medical professionals. By addressing these challenges, Iraq could retain its neurosurgeons and create a more sustainable healthcare system that serves the needs of its population.

The action for building endovascular centers

The establishment of endovascular centers requires significant investment in infrastructure, including state-of-the-art angiography suites and advanced medical imaging equipment. These centers must also be staffed with highly trained professionals, such as neurosurgeons and interventional radiologists, who can perform the intricate procedures necessary for stroke treatment. Training programs are essential for equipping local practitioners with the skills required to operate these advanced facilities, particularly as Iraq builds a foundation for rapid-response stroke care networks [2,5]. Furthermore, collaboration with international medical organizations and universities could facilitate knowledge-sharing and provide training opportunities for Iraqi neurosurgeons. This investment in both infrastructure and professional development would enable Iraq to establish a high-quality, sustainable stroke treatment network, enhancing the healthcare system’s capacity to respond to neurological emergencies.

Implementing telemedicine solutions could extend the reach of endovascular centers, ensuring that patients in rural or underserved areas have access to specialized stroke care. Telemedicine can be used to provide remote consultations, allowing doctors in local hospitals to connect with specialists in centralized stroke centers. This model has proven effective in other regions, where telemedicine networks ensure patients in remote locations receive timely and appropriate care through coordinated transfers and guidance from specialists. In Iraq, a similar system could connect smaller hospitals to centralized endovascular centers, ensuring more equitable access to advanced care for stroke patients.

By integrating telemedicine into Iraq’s healthcare strategy, the country could expand the impact of its endovascular centers, reaching more patients and reducing disparities in access to life-saving treatments.

Endovascular treatment for stroke sits at a crucial intersection of neurosurgery, neurology, and radiology, yet significant training gaps among neurologists and radiologists limit its effective implementation in Iraq. While neurosurgeons are more familiar with invasive procedures, neurologists and radiologists often lack specialized training in endovascular techniques. This shortfall in expertise reduces the efficiency and collaborative potential needed for timely stroke interventions, hindering Iraq’s ability to establish high-functioning endovascular centers.

Addressing these training deficits is essential to the success of endovascular centers. Establishing dedicated training programs and partnerships with international institutions could empower neurologists and radiologists to actively support endovascular stroke care, while in-country fellowships or workshops could provide hands-on experience. Such initiatives would build a well-rounded, multidisciplinary workforce, ensuring that Iraq’s endovascular centers are not only feasible but also sustainable in delivering improved stroke outcomes and aligning with international standards [1,2,5].

Conclusion

Building endovascular centers in Iraq is essential to address the escalating burden of stroke and improve patient outcomes nationwide. In Iraq's unique socio-political environment, such centers are not only a medical necessity but a lifeline for patients facing severe neurological emergencies. Addressing these needs through endovascular treatment centers would align Iraq’s healthcare infrastructure with global standards, ensuring that more lives are saved, and long-term disabilities are minimized. By focusing on infrastructure development, professional training, and telemedicine integration, Iraq can establish a comprehensive stroke care framework, support its healthcare professionals, and reduce the societal burden associated with stroke. This coordinated approach offers the potential to significantly improve the quality and accessibility of stroke care in Iraq.
